# High-Level Cross-Resistance to Didanosine Observed in South African Children Failing an Abacavir- or Stavudine-Based 1^st^-Line Regimen

**DOI:** 10.1371/journal.pone.0097067

**Published:** 2014-05-09

**Authors:** Kim Steegen, Leon Levin, Irene Ketseoglou, Michelle Bronze, Maria A. Papathanasopoulos, Sergio Carmona, Wendy Stevens

**Affiliations:** 1 Department of Haematology and Molecular Medicine, University of Witwatersrand, Johannesburg, South Africa; 2 National Health Laboratory Services, Johannesburg, South Africa; 3 Right to Care, Johannesburg, South Africa; University of California, San Francisco, United States of America

## Abstract

**Background:**

The knowledge-base of emerging drug resistance profiles in children exposed to abacavir-based antiretroviral regimens in South Africa is very limited. This study investigated the suitability of didanosine-based 2^nd^-line regimens for children in the context of antiretroviral drug resistance patterns emerging after 1^st^-line virologic failure.

**Methods:**

A retrospective dataset of 354 antiretroviral drug resistant genotypes from children failing either abacavir (n = 81) or stavudine (n = 273) based 1^st^-line regimens, was analysed. Samples were sent to the HIV genotyping laboratory at Charlotte Maxeke Johannesburg Academic Hospital, for routine testing. P*ol* sequences were submitted to the Stanford HIV drug resistance database for genotypic predictions.

**Results:**

Children were exposed to abacavir or stavudine-based 1^st^-line regimens for an average of 21 and 36 months, respectively. The frequency of reduced susceptibility to didanosine was substantial in the abacavir-exposed group (69.1%).This reduced susceptibility was commonly attributed to L74V/I (n = 44) and to a lesser extent K65R (n = 10) mutations. Didanosine resistance was observed in 43.2% of patients exposed to stavudine-based regimens. In contrast, most children remained susceptible to stavudine regardless of exposure to abacavir (77.8%) or stavudine (74.7%). At least 80% of children remained susceptible to zidovudine irrespective of stavudine or abacavir-exposure. The presence of the K65R mutation was more common after abacavir pressure (12.3% vs 1.8%).

**Conclusion:**

Analysis revealed that didanosine-based 2^nd^-line regimens have limitations for South African children, given the high frequency of mutations that confer cross-resistance to didanosine; especially after abacavir-exposure. This data has influenced South African paediatric treatment guidelines, which now recommend zidovudine-based 2^nd^-line regimens.

## Introduction

By the end of 2011 an estimated 460 000 South African children were infected with Human Immunodeficiency Virus type 1 (HIV-1) [1], of which only 152 000 were on antiretroviral drug treatment (ART) [2]. Since August 2012, the National Department of Health recommends ART initiation in all children under 5 years of age, regardless of their immunological or clinical status [3].

The early initiation of treatment has reduced early mortality in the paediatric population [4], but has led to the inevitable increase in the number of children failing ART and subsequent development of HIV-1 drug resistance. Since children face life-long treatment ahead of them, it should be ensured that the available 1^st^ and 2^nd^-line regimens are maintained as long as possible. Recently, several studies addressed the HIV-1 drug resistance profiles in South African children failing ART [5]–[9]. These studies noted very similar reverse transcriptase (RT) resistance patterns as compared to adults, with M184V and K103N being the most prevalent mutations [5], [6], [9]. Conversely, various studies highlighted a much higher prevalence of protease inhibitor (PI) resistance mutations compared to adults [6]–[9]. This difference in PI resistance might partly be explained by the single-dose ritonavir exposure in a significant number of children. Yet, none of these studies described the HIV-1 drug resistance profile of abacavir (ABC) based regimens.

In 2010, the South African preferred 1^st^-line paediatric regimen was changed from a stavudine and lamivudine (d4T+3TC) based regimen to an ABC and 3TC-based regimen, with a suggested switch to a didanosine and zidovudine (ddI+AZT) based 2^nd^-line regimen upon virological failure [10].

In view of the lack of evidence about the HIV-1 drug resistance profile after ABC-exposure in a South African setting, and with the understanding that mutations arising from the use of d4T and ABC can confer cross-resistance to ddI, a retrospective study was conducted to investigate the HIV-1 drug resistance profiles in children failing d4T or ABC-based 1^st^-line regimens and the subsequent implications for a ddI-based 2^nd^-line regimen.

## Materials and Methods

### Ethics statement

This study was conducted with Ethical clearance by the Research on Human Subjects (Medical) Committee at the University of the Witwatersrand (Clearance Number M120730). Due to the retrospective nature of this study, no informed consent was obtained from the next of kin, caretakers, or guardians on behalf of the children. This is in line with the Research on Human Subjects (Medical) Committee policy which states informed consent is not required for this type of study and a waiver was hence granted.

### Patient samples

At the time of data collection, the National Health Laboratory Service (NHLS) offered HIV drug resistance testing at two laboratories in South Africa (Johannesburg and Stellenbosh). The HIV Genotyping Laboratory at Charlotte Maxeke Johannesburg Academic Hospital accepts and processes samples for HIV drug resistance testing from any government health facility in the country, but most samples are collected from Gauteng, North West and KwaZulu-Natal provinces. Due to the lack of specific HIV-1 drug resistance testing guidelines, at the time of study, the patients referred for resistance testing are identified at the clinicians' discretion. However, all patients need to have proof of virological failure, which is defined as at least one HIV viral load >1000 copies/ml. All sequences obtained from The HIV Genotyping Laboratory at Charlotte Maxeke Johannesburg Academic Hospital are stored in a database (TherapyEdge, Advanced Biological Laboratories, South Africa).

For this analysis, only sequences obtained at the HIV Genotyping Laboratory at Charlotte Maxeke Johannesburg Academic Hospital from children ≤15 years old with known treatment history, who were exposed to a d4T+3TC (n = 279) or ABC+3TC-based regimen (n = 91) at the time of HIV drug resistance testing were included in this study. Children who had prior exposure to any other ART regimen were not excluded from analysis. The data retrieval was limited to sequences obtained from samples collected between 2009 and 2012. Demographic and clinical information such as age, ART regimen, time on ART, and the latest viral load measurements, was collected from laboratory request forms or the electronic laboratory information system. Phylogenetic analysis (Mega5 [11]) and demographic information was used to ensure that only one sample per patient was included in the analysis.

### Pol genotyping

The protease (PR) and reverse transcriptase (RT) nucleotide sequences were obtained using the ViroSeq HIV-1 Genotyping Assay (Abbott Molecular, Illinois, USA) or the in-house genotyping protocol [12]–[14]. Both assays have a lower limit of detection of 1000 copies/ml. The in-house protocol was extensively validated against the ViroSeq HIV-1 Genotyping Assay and results were proven to be equivalent.

### Resistance mutation analysis

The retrieved sequences were resubmitted to the ViroScore database (TherapyEdge, Advanced Biological Laboratories, South Africa) for updated analysis with the Stanford HIV-1 database v6.2.0. The identification of the HIV-1 drug resistance mutations was based on the most recent International AIDS Society mutation list [15]. Predicted antiretroviral drug resistance profiles were categorized as either being susceptible (including potential low-level resistance), intermediate (including low-level resistance) or high-level resistant. All samples were subtyped using the REGA subtyping tool v2.0 [16].

### Statistical analysis

Statistical analyses were performed using GraphPad Prism 6. Differences between groups for non-parametric data such as age, viral load and time on treatment were calculated using the 2-sided Wilcoxon Mann-Witney test. Two-sided Fisher's exact tests were performed to assess proportional differences between groups for gender and mutation prevalence. A p-value of <0.05 was considered statistically significant. Due to the retrospective nature of this analysis, some clinical and demographical data was not available for analysis. The health care facility was not contacted to obtain these missing clinical or demographic data. Since the missing data was completely random, the dataset was analysed as such, without taking missing data into account.

### Sequence data

The *pol* nucleotide sequences were submitted to GenBank using Sequin v9.50 (www.ncbi.nlm.nih.gov/Sequin) and are available under accession numbers KJ176285 to KJ176654.

## Results

Three-hundred and seventy (n = 370) appropriate HIV-1 drug resistance results were retrieved from the database. Patient demographics and clinical data are summarized in Table 1. Male children (55.1%) were slightly better represented in the dataset compared to female counterparts. The majority of children (88.6%) were between 3 and 15 years old. The average age of children, known to be (n = 93) or have been (n = 16) exposed to PI-containing regimens was significantly younger compared to those children without exposure to PIs (5 versus 10 years respectively, p<0.0001). This finding confirms adherence to the treatment guidelines, which recommend children younger than 3 years of age to be initiated on a PI-based regimen. The median viral load of all children included in this analysis was 4.6 log RNA copies/ml after a median treatment duration of 28 months (Table 1).

**Table 1:** Demographics and clinical characteristics of 370 South African children failing a 1^st^-line antiretroviral drug regimen.

		Patient demographics								
	All patients (n = 370)	ABC-group (n = 91)	d4T-group (n = 279)							
	N (%)	Mean	IQR	N (%)	Mean	IQR	N (%)	Mean	IQR	p-value
**Gender**										
*F*	161 (43.5)			43 (47.3)			118 (42.3)			0.1440
*M*	204 (55.1)			46 (50.5)			158 (56.6)			
*unknown*	5 (1.4)			2 (2.2)			3 (1.1)			
**Age** *(years)*	366 (98.9)	8	5–12	90 (98.9)	8	3–12	276 (98.9)	9	5–12	0.4235
**Viral Load** *(log RNA copies/ml)*	341 (92.2)	4.6	4.0–5.1	86 (94.5)	4.7	4.2–5.4	255 (91.4)	4.5	3.9–5.0	0.0291
**Time on ART regimen** *(months)*										
Time on current regimen	282 (76.2)	31	18–47	65 (71.4)	18	12–30	217 (77.8)	34	22–50	<0.0001
Time on previous regimen	55 (14.9)	24	16–36	38 (46.9)	24	21–35	17 (6.1)	18	11–38	0.1227
Total time on ART	282 (76.2)	35	21–50	65 (71.4)	25	14–45	217 (77.8)	35	24–52	0.0115
**HIV drug resistance detected**	354 (95.7)			81 (89.0)			273 (97.8)			0.0011

Details about the antiretroviral drug treatment history of all children are depicted in Table 2. Most children (n = 279, 75.4%) were failing a d4T-containing regimen, after median time of exposure to that regimen of 34 months (IQR: 21–50 months). Seventeen (6.1%) of these children had prior ART exposure for a median time of 18 months. An additional 91 children (24.6%) were failing ABC-based regimens at the time of HIV drug resistance testing after a median of 18 months (IQR 12–24 months) exposure to the current regimen. However, 38 children in the ABC-group had documented prior ART exposure (median 24 months, IQR 19–36 months). This prior ART exposure included d4T for 34 (41.9%) children as their treatment was most likely changed when the new treatment guidelines were released in 2010. The effect of prior exposure to d4T in the ABC-group was assessed by comparing the prevalence of each mutation between the children who had only been exposed to ABC-based regimens (n = 47), versus those who were currently failing and ABC-based regimen, but had prior exposure to d4T (n = 34). The proportion of these mutations did not significantly differ between the two groups (data not shown), therefore all 81 children failing an ABC-base regimen at the time of HIV drug resistance testing were analysed as one group. Analysis of the antiretroviral drug resistance profiles showed that 354 children harboured virus with known drug resistance mutations. Fully susceptible drug resistance profiles were only observed in 4.3% (n = 16) of the examined population. This observation was significantly more common in the ABC-exposed group (n = 10, 11.0%) versus the d4T group (n = 6, 2.1%, p = 0.0011). These 16 “wild-type” genotypes were excluded from subsequent analysis. Subtyping revealed that most children were infected with HIV-1 subtype C. One child was infected with a subtype B virus and two others were infected with an A/C recombinant virus.

**Table 2:** Antiretroviral Treatment history of 370 children failing a 1^st^-line antiretroviral drug regimen.

ART at time of virological failure	Number of patients N (%)	Previous ART regimen^a^	Number of patients N
**ABC-based**	**91 (24.6)**		
ABC+3TC+EFV	57 (15.4)	**d4T**+3TC+EFV	21
		**d4T**+3TC+**NVP**	1
		**d4T**+3TC+**LPV/r**	1
		**d4T**+3TC+ABC+**LPV/r**	1
		**d4T**	1
		AZT+3TC+**LPV/r**	1
		ABC+3TC+**TDF**	1
		none	30
ABC+3TC+LPV/r	34 (9.2)	**d4T**+3TC+LPV/r	5
		**d4T**+3TC+**EFV**	3^b^
		**d4T**+AZT+3TC+LPV/r	1
		ABC+3TC+**EFV**	1
		**NVP**	1
		none	23
**d4T-based**	**279 (75.4)**		
d4T+3TC+EFV	190 (51.4)	d4T+3TC+**LPV/r**	12
		d4T+3TC+**NVP**	1
		**AZT**+3TC+**NVP**	1
		none	176
d4T+3TC+LPV/r	70 (18.9)	d4T+3TC+**EFV**	2
		none	68
d4T+3TC+NVP	14 (3.8)	d4T+3TC+**LPV/r**	1
		none	13
d4T+3TC+TDF	4 (1.1)	none	4
d4T+3TC+SQV/r	1 (0.3)	none	1

a Abbreviations: ABC: abacavir, TDF: tenofovir, ddI: didanosine, d4T: stavudine, 3TC: lamivudine, AZT: zidovudine, NVP: nevirapine, EFV: efavirenz, LPV/r: boosted lopinavir, SQV/r: boosted saquinavir.

b One patient had prior exposure to AZT+ddI+LPV/r.

**Figure 1:**
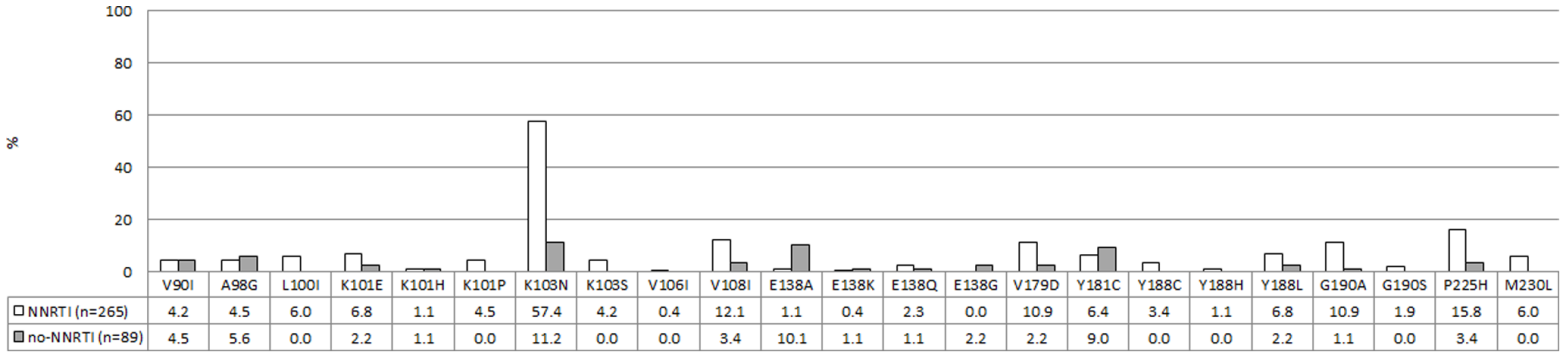
Non-nucleoside reverse transcriptase inhibitors (NNRTI) mutation profiles. Profiles associated with failure to NNRTIs are depicted in white, profiles associated with failure to non-NNRTI regimens are depicted in grey. The numbers represent percentages.

**Figure 2:**
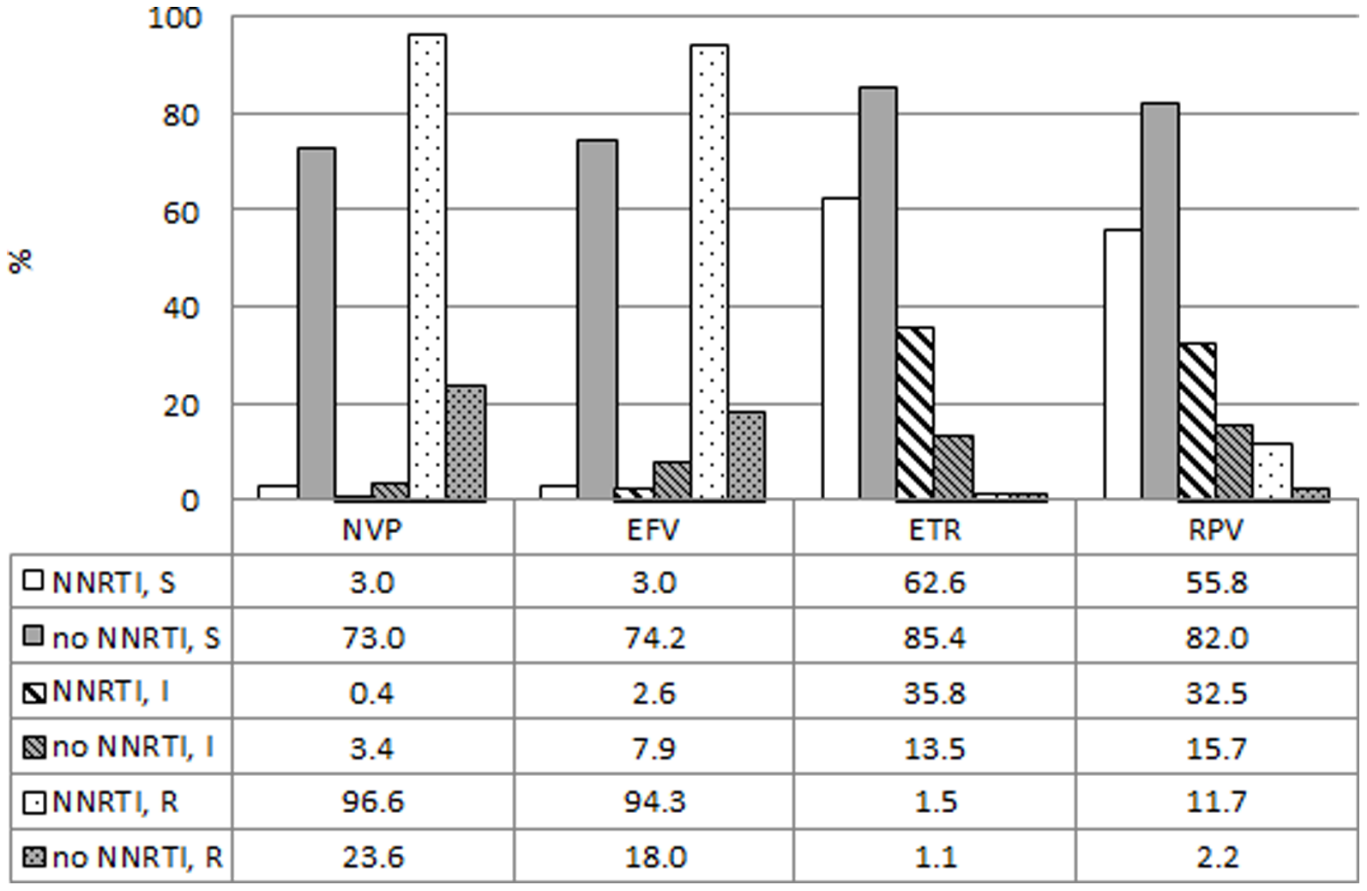
Prevalence of resistance to non-nucleoside reverse transcriptase inhibitors. Prevalence of susceptible (S, solid bars), intermediate (I, striped bars) or high-level resistance (R, dotted bars) to non-nucleoside reverse transcriptase inhibitors (NNRTIs) at failure of an NNRTI-based (n = 265, white) or non-NNRTI-based regimen (n = 89, grey). Eight (n = 8, 8.2%) patients included in the NNRTI group were only exposed to an NNRTI during a previous regimen. The numbers represent percentages. Abbreviations: NVP: nevirapine, EFV: efavirenz, ETR: etravirine, RPV: rilpivirine.

A detailed analysis of mutations associated with NNRTI resistance stratified according to a NNRTI or non-NNRTI-containing regimen, is depicted in Figure 1 and 2. A total of 265 children (74.9%) had been exposed to EFV or NVP, including 8 children who received a LPV/r-based regimen at time of resistance testing. The most common NNRTI mutation in this group was K103N (n = 152, 57.4%) followed by V106M (n = 105, 39.6%), P225H (n = 42, 15.8%) and V179D (n = 29, 10.9%, Figure 1). The Y181C mutation appeared more frequently among children without recorded NNRTI-exposure (9.0% vs 6.4%), this trend was however not statistically significant (p = 0.4728). The presence of other NNRTI-mutations such as K103N (11.2%) in the non-NNRTI group indicates that non-disclosed NNRTI-exposure might have taken place in some of these patients, most likely as a result of PMTCT. Predictably, most viruses from NNRTI-exposed children showed high-level resistance to NVP (96.6%) and EFV (94.3%). Of note, more than a third of the children in this group showed reduced susceptibility to 2^nd^-generation NNRTIs such as etravirine (37.3%) and rilpivirine (44.2%, Figure 2).

**Figure 3:**
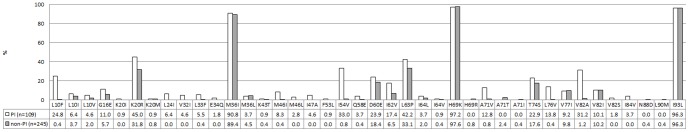
Protease Inhibitor mutation profiles. Mutation profiles associated with failure to PIs are depicted in white, mutation profiles associated with failure to non-PI-based regimens are depicted in grey. The numbers represent percentages.

**Figure 4:**
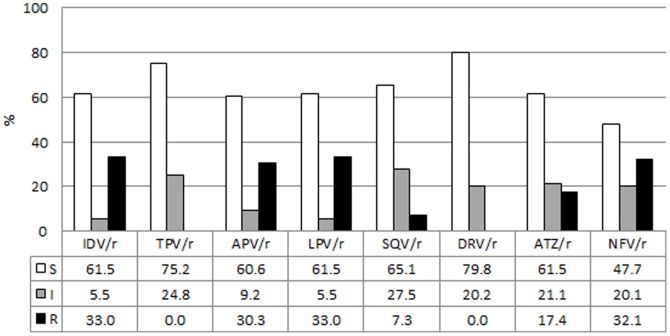
Prevalence of resistance to protease inhibitors. Prevalence of susceptible (S, white), intermediate (I, grey) or high-level resistance (R, black) to PIs at failure of a PI-based (n = 93) regimen. Fifteen (15) children failing NNRTI-based regimens, with known prior exposure to PIs were included in this group. The numbers represent percentages. Abbreviations: IDV/r: boosted indinavir, TPV/r: boosted tipranavir, APV/r: boosted fosamprenavir, LPV/r: boosted lopinavir, SQV/r: boosted saquinavir, DRV/r: boosted darunavir, ATZ/r: boosted atazanavir, NFV/r: boosted nelfinavir.

A detailed analysis of mutations associated with PI resistance, stratified according to a PI or non-PI-containing regimen, is depicted in Figure 3 and 4. Most children (n = 245, 69.2%) did not have any documented exposure to protease inhibitors, hence 80.8% (n = 198) did not show any level of resistance to this drug class (Figure 3). Of the children without documented PI-exposure, 44 (18.0%) children showed reduced susceptibility to boosted nelfinavir (NFV/r) (Figure 4), caused by polymorphism T74S mutation in all but one child (Figure 3).This T74S mutation is a known subtype C polymorphism. Reduced susceptibility to NFV/r in the remaining patient was caused by N88D. A further three children, without documented PI-exposure, presented with medium to extensive PI resistance. One patient showed intermediate resistance to 4 PIs, including LPV/r. Another patient presented with high-level resistance to 4 PIs, including LPV/r, as well as intermediate resistance to 3 PIs. The third patient showed high-level resistance to all PIs except boosted tipranavir (intermediate resistance) and boosted darunavir (susceptible). The resistance profiles of these three patients indicate either undisclosed PI-exposure or transmitted drug resistance.

One third of the children in the study (n = 108, 30.5%) had documented exposure to LPV/r, including 16 children who were exposed to LPV/r at an earlier stage during treatment. In addition, one child failed a boosted saquinavir-based regimen. Almost half of the children in the PI-exposed group (n = 52, 47.7%) did not harbour any PI-related mutations. Fourteen children (12.8%) harboured virus with reduced susceptibility to NFV/r due to the T74S polymorphism in 13 patients, and K20T in one child. Three children showed intermediate resistance to two, five or seven PIs. The remaining 40 children (36.7%) presented with high-level resistance to at least one PI in combination with various levels of intermediate resistance to other PIs. Thirty-five of these children showed high-level resistance to at least three PIs.

**Figure 5:**
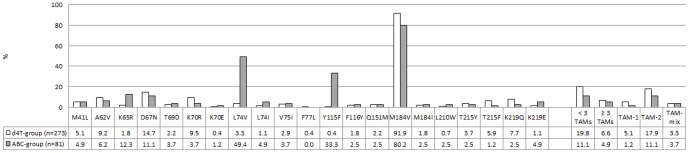
Nucleoside/tide Reverse Transcriptase Inhibitor (NRTI) mutation profiles. Nucleoside/tide Reverse Transcriptase Inhibitor (NRTI) mutation profiles associated with failure to d4T- or ABC -based regimens are depicted in white and grey respectively. The numbers represent percentages.

**Figure 6:**
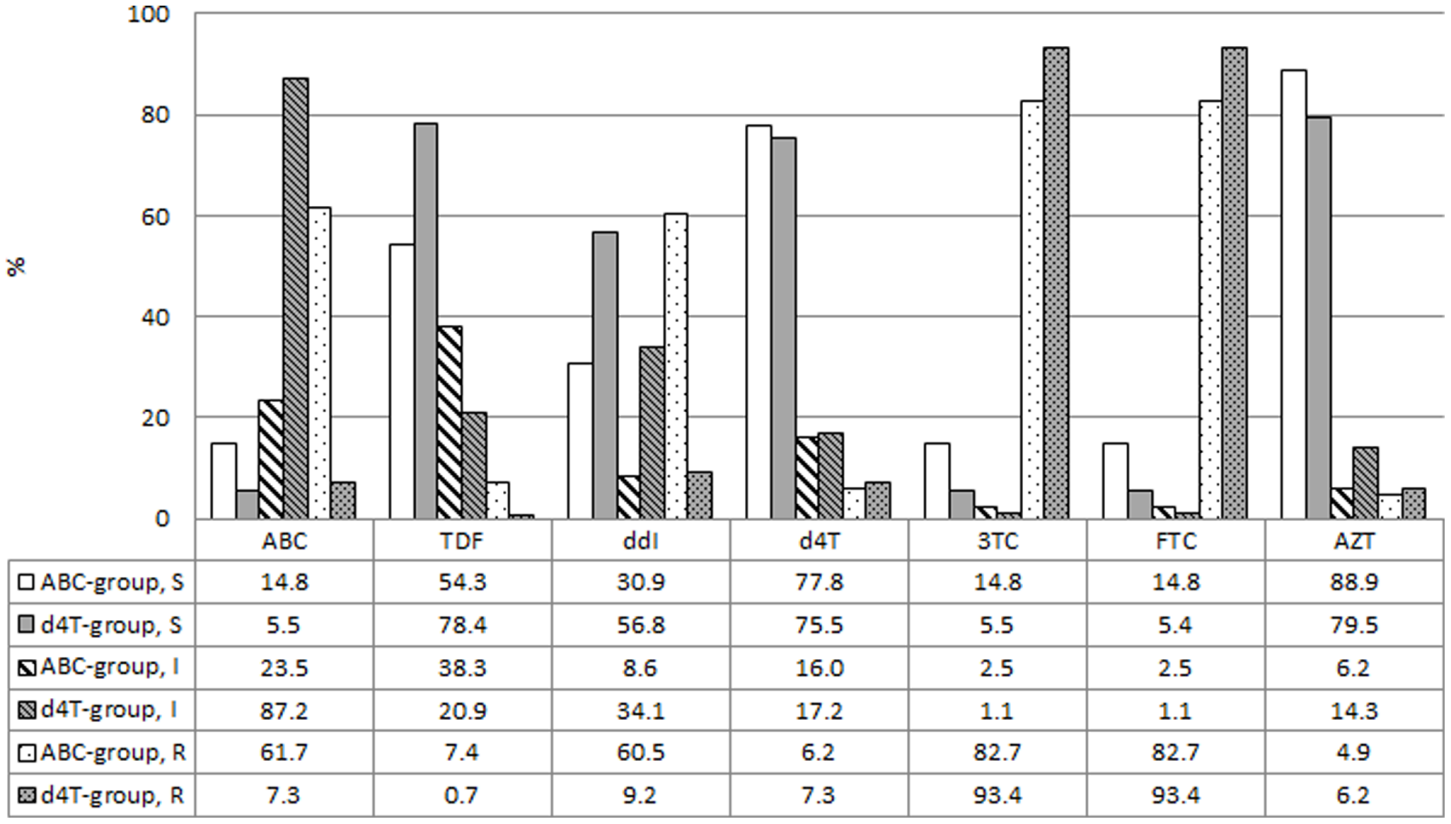
Prevalence of resistance to nucleoside/tide Reverse Transcriptase Inhibitors. Prevalence of susceptible (S, solid bars), intermediate (I, striped bars) or high-level resistance (R, dotted bars) to nucleoside reverse transcriptase inhibitors (NRTIs) after exposure to an ABC containing regimen (n = 81, white) or d4T containing regimen (n = 273, grey). The numbers represent percentages. Abbreviations: ABC: abacavir, TDF: tenofovir, ddI: didanosine, d4T: stavudine, 3TC: lamivudine, FTC: emtricitabine, AZT: zidovudine.

All children were exposed to 3TC, and the majority of them (n = 316, 89.3%) harboured the M184V/I mutation (Figure 5). This mutation contributed to high-level resistance to 3TC/FTC in the d4T (n = 255, 93.4%) and ABC-exposed group (n = 67, 82.7%, Figure 6). At time of failure, children exposed to ABC demonstrated high-level resistance to ABC (n = 50, 61.7%), while an additional 19 children (23.5%) presented with intermediate resistance to ABC (n = 19, 23.5%). The presence of high-level and intermediate cross-resistance to ABC in the d4T-exposed group was observed in 22 (8.1%) and 235 (86.1%) children, respectively (Figure 3B). A large proportion of children remained susceptible to d4T regardless of exposure to ABC (n = 63, 77.8%) or d4T (n = 204, 74.7%, p = 0.6601, Figure 6). Most children remained fully susceptible to AZT after exposure to ABC (n = 72, 88.9%) and d4T (n = 217, 79.5%, p = 0.0712). The prevalence of complete susceptibility to TDF was significantly larger in the d4T group (n = 214, 78.4%) compared to the ABC group (n = 44, 54.3%, p<0.0001). However, only 6 children (7.4%) from the ABC group showed high-level resistance to ABC. The frequency of reduced susceptibility to ddI was substantial in the ABC-exposed group. Forty-nine (60.5%) children presented with high-level resistance and another and 7 children (8.6%) showed intermediate resistance to ddI (Figure 6). Resistance to ddI was commonly attributed to the L74V/I mutation (n = 44). Despite the lower rate of high-level ddI resistance after exposure to d4T (n = 25, 9.2%) an important proportion of these patients still showed intermediate resistance to ddI (n = 91, 33.3%, Figure 6).

The presence of thymidine analogue mutations (TAMs) did not seem to be affected by the nucleoside backbone with 19.8% (n = 54) of d4T-exposed children having ≤2 TAMs compared to 11.1% (n = 9) in the ABC-exposed group (p = 0.0970). The detection of ≥3 TAMs remained limited to 4.9% (n = 4) and 6.6% (n = 18) of the patients in the ABC and d4T group, respectively (p = 0.7942). Seven of the 13 children who presented with TAMs after ABC-exposure were known to have received prior treatment containing d4T; in three of them more than 3 TAMs were detected. The preferred TAM-2 pathway was the most common pathway in both groups (Figure 5). K65R was more common among children failing ABC-based regimens (n = 10, 12.3%) compared to the d4T-exposed group (n = 5, 1.8%, p = 0.0003). The Q151M mutation was only detected in 2 ABC-exposed children (2.5%) and 6 d4T-exposed children (2.2%).

## Discussion

This retrospective study provides important insights into the HIV-1 antiretroviral drug resistance profile in South African children failing prevailing 1^st^-line ART regimens. Due to the change in the South African paediatric ART guidelines in 2010 [10] we were able to compare resistance profiles from children suspected to fail d4T or ABC-based regimens. Overall, only a small subset of children (4.3%) did not have any HIV-1 drug resistance mutations, which is comparable to findings in some South African studies [5], [9], but lower than other studies that showed a prevalence of 19–22% of wild type viruses [6], [7]. Since the absence of drug resistance mutations in children failing ART is likely due to poor compliance, these differences between the studies can be attributed to various adherence levels between the cohorts.

More than 95% of children, exposed to NNRTIs, showed resistance to NVP and EFV, which is higher compared to the findings from other studies [5], [6], [9]. Almost a quarter of the children without recorded exposure to NNRTIs also presented with resistance to NVP and EFV. This outcome might be explained by undisclosed exposure to NNRTIs, either due to prior treatment with NNRTI-containing regimens or the use of NNRTI-containing PMTCT-regimens. Alternatively NNRTI resistance can be attributed to vertical transmission of antiretroviral drug resistant virus. The observation of reduced susceptibility to 2^nd^-generation NNRTIs in one third of the patients is of concern, since it might jeopardize the use of these drugs in 3^rd^-line regimens.

More than 60% of the children exposed to PIs did not show any PI-mutation, other than the T74S polymorphism. This polymorphism is prevalent in 10% of subtype C wild type viruses [17]. The prevalence of PI resistance in our study (30.5%) was very similar to that found by others where the prevalence ranged from 36 to 44% [7], [9]. The three children who presented with PI resistance, without documented exposure to PIs, have likely not disclosed previous treatment with PIs. It is of concern that those children, who do present with PI-resistance, showed extensive levels of resistance which significantly jeopardizes future treatment options. Having said this, it is unknown, how many of these children had prior exposure to full dose ritonavir, which is known to cause more extensive PI resistance [8].

The frequency of M184V/I and consequent high-level resistance to 3TC/FTC is consistent with results found in most other South African studies [5], [7], [9]. However, this finding does not significantly impact options for 2^nd^-line drugs, as M184V/I reduces viral fitness [18] and often delays the development of TAMs [19]. This M184V/I mutation also caused intermediate resistance to ABC in more than 80% of the d4T-exposed children. Surprisingly about 75% of children remained susceptible to d4T regardless of exposure to d4T or ABC. This finding suggests that d4T or AZT can be viable options in 2^nd^-line regimens. Due to its toxic effects, the use of d4T in its current dosage is no longer recommended by the WHO [20]. Nevertheless, if toxicity can be reduced by lowering the dosage, d4T could still be a feasible option in resource-limited settings, both for children and adults [21]. A clinical trial to investigate this option in adults is currently on-going in South Africa, Uganda and India (http://www.wrhi.ac.za/Pages/ClinicalTrials.aspx). The K65R mutation was more often present in ABC-exposed children compared to those exposed to d4T, which has recently been described by Van Zyl *et al*. [22]. The development of TAMs is known to be more prevalent under thymidine analogues, but the higher prevalence of TAMs in the d4T-exposed group might also be explained by the longer treatment duration in this group. Both the presence of TAMs and K65R has an impact on the NRTI options available for 2^nd^ and 3^rd^-line regimens. Due to the relatively low prevalence of both TAMs and K65R, the majority of children remain susceptible to AZT and TDF.

The most significant finding however is the increased frequency of ddI cross-resistance seen in children exposed to ABC. More than 60% of these children showed high-level cross-resistance to ddI, which was a component of the 2^nd^-line regimen in South Africa since 2010 [10]. The frequent detection of resistance to ddI, major toxicity issues and the difficult dosing schedule discourage the use of ddI. Our findings strongly suggest excluding ddI from the 2^nd^-line paediatric regimen and replacing it by AZT and recycled 3TC as our data indicates that 88.9% of patients failing ABC-based regimens remain susceptible to AZT. This recommendation has shaped the new guidelines for paediatric antiretroviral treatment in South Africa where an AZT and 3TC now form the 2^nd^-line NRTI backbone for children failing ABC-based regimens [23].

This study has some limitations, in that it is a retrospective analysis of routine laboratory data with limited clinical data. For those children who had prior ART exposure, the reason for switch or the viral load at time of switch was unknown. Occasionally, antiretroviral treatment history might have been incorrect or incomplete, which might have led, in some cases, to incorrect categorization of children as per their ART exposure. In the absence of national HIV-1 drug resistance testing guidelines, the dataset may be biased towards sampling children under the care of clinicians reliant on HIV-1 drug resistance testing for clinical management and may not provide proportional representation of children failing ART in South Africa. However, these disadvantages are largely overcome by the large sample size of the study.

To our knowledge this is the first report that compares the HIV-1 antiretroviral drug resistance profiles between children exposed to d4T and ABC-containing 1^st^-line regimens. The findings reported here influenced the design of the 2013 South African ART guidelines and ensured ddI is no longer a recommended antiretroviral agent for the treatment of HIV-infected children in South Africa. The complex mutation profiles observed in children failing NNRTI and especially PI-based regimens, suggest that HIV-1 drug resistance testing is beneficial in the paediatric population.
